# Chronic Expanding Hematoma in the Extremities: A Clinical Problem of Adhesion to the Surrounding Tissues

**DOI:** 10.1155/2017/4634350

**Published:** 2017-05-31

**Authors:** Akio Sakamoto, Takeshi Okamoto, Shuichi Matsuda

**Affiliations:** Department of Orthopaedic Surgery, Graduate School of Medicine, Kyoto University, Shogoin, Kawahara-cho 54, Sakyo-ku, Kyoto 606-8507, Japan

## Abstract

Chronic expanding hematoma is characterized by continuous growth of a blood collection. We analyzed the clinical features of 7 patients with chronic expanding hematomas in the extremities, with an average age of 65.6 years. All lesions occurred in the lower extremities, with 4 seen in the thigh and 3 in the knee region. Six patients had subcutaneous hematomas, while 1 was deep-seated in the thigh. The magnetic resonance features of the lesion were compatible with those of a standard hematoma. A low signal intensity on T1- and T2-weighted imaging at the pseudocapsule was also characteristic. Cystic features were seen in 5 of 7 patients. All lesions were resected together with their pseudocapsule. In the subcutaneous lesions, it was necessary to resect adherent fascia, with or without involved skin. In the deep-seated thigh lesion, the common peroneal nerve was completely adherent to the pseudocapsule, a phenomenon from absence of the common peroneal nerve which appeared after resection. Chronic expanding hematomas of the extremities are predominantly located in the subcutaneous tissue of the lower extremity. The surrounding pseudocapsule is adherent to the adjacent tissues, and clinicians must be aware of this, especially when resecting a deep-seated lesion.

## 1. Introduction

Hematomas are usually reabsorbed, slowly decreasing in size over time. In rare cases, hematomas slowly increase in size and are then referred to as chronic expanding hematomas [1, 2]. The chronic expanding hematoma is characterized by a mixture of old and new blood, accompanied by necrotic degradation and liquefaction and a fibrous pseudocapsule [2]. An association with trauma has been reported [2]. Chronic expanding hematomas occur in various locations [1, 2]. In this report, we analyze patients with chronic expanding hematomas of the extremities.

## 2. Case Series

**Figure 1:**
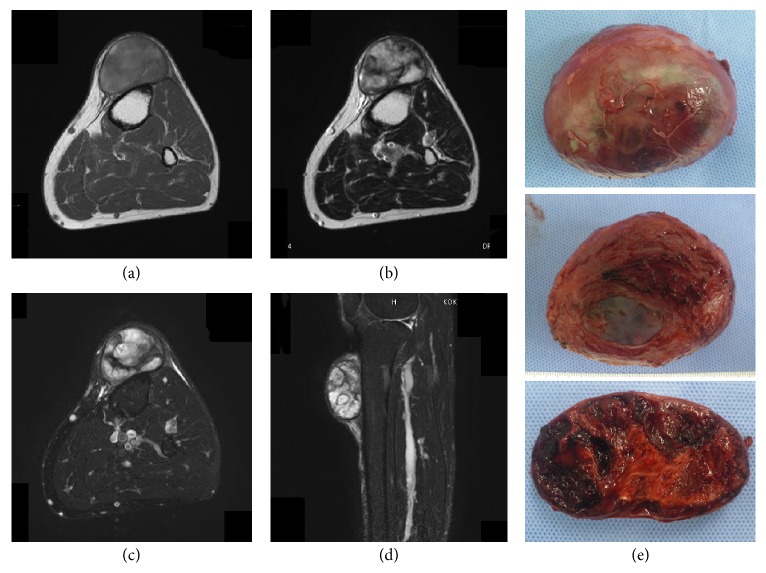
67-year-old male with a chronic expanding hematoma in the subcutaneous tissue of the knee region. Magnetic resonance imaging (MRI) shows heterogeneous low to intermediate signal intensity on T1-weighted imaging (a) and heterogeneous low to high signal intensity on T2-weighted imaging (b). Fat-suppressed T2-weighted imaging fails to suppress the high signal-intensity area seen in (c, d) ((a–c) axial image; (d) sagittal image). The resected specimen is seen from the top surface (upper), bottom surface (middle), and cut surface (lower); hemorrhagic coagulation is visible (e).

**Figure 2:**
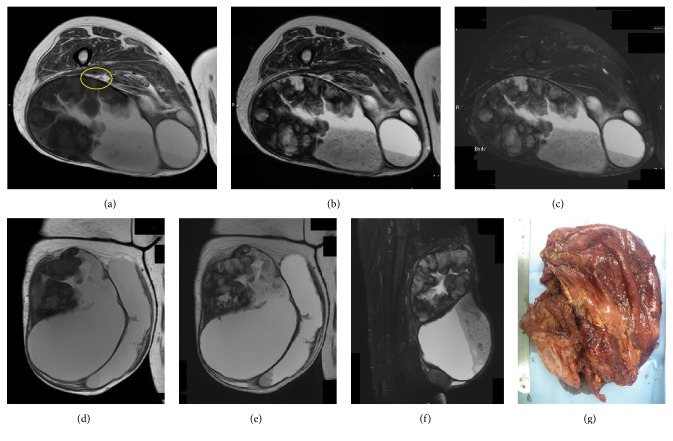
69-year-old female with a chronic expanding hematoma in the deep thigh. MRI shows a huge lesion with cystic features ((a–c) axial image; (d-e) coronal image; (f) sagittal image). The sciatic nerve where it branches into the tibial nerve and the common peroneal nerve is not obvious on axial section. A yellow circle indicates the area where the nerve is supposed to exist (a). MRI shows heterogeneous low to slightly intermediate intensity on T1-weighted imaging (a, d). Heterogeneous low to high signal intensity is seen on T2-weighted (b, e) and fat-suppression T2-weighted imaging (c, f). A fibrous cystic wall is visible after removal of the hemorrhagic coagulation material (g).

The clinical data are summarized in Table 1. In 7 patients with chronic expanding hematomas in the extremities, 6 were male and 1 was female. The mean age at the time of resection was 65.6 years, ranging from 54 to 79 years. The location of the chronic expanding hematoma was in the lower extremity in all patients: the thigh was involved in 4 patients and the knee region in 3 patients. Six lesions occurred in the subcutaneous tissue (Figure 1), while 1 occurred in the deeper tissue of the thigh (Figure 2). Laboratory examination, including C-reactive protein levels, revealed no remarkable findings. A history of trauma was present in 2 of the 7 patients, and only a single patient was taking anticoagulation medication.

**Figure 3:**
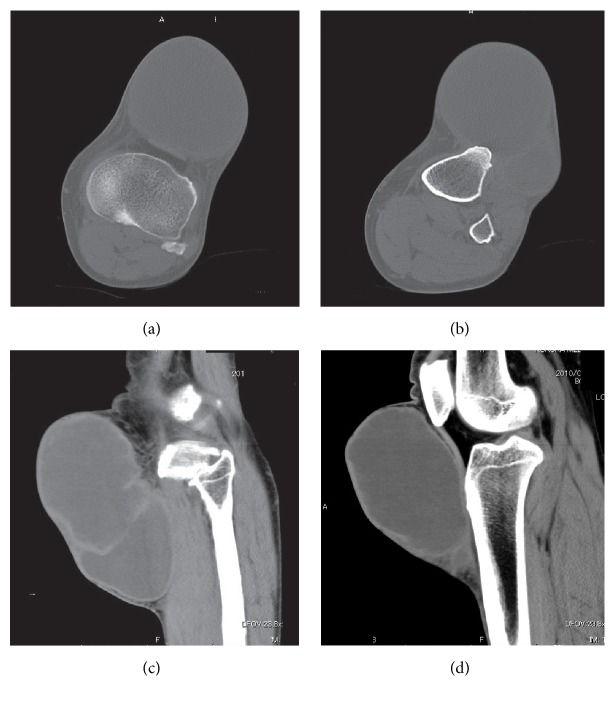
61-year-old male with a subcutaneous chronic expanding hematoma in the knee region. Computed tomography shows a cystic lesion with a pseudocapsule and a septum. Subcutaneous fat tissue is not observed between the lesion and the overlying skin ((a-b) axial bone window; (c-d) sagittal soft-tissue window).

Patients were evaluated using either magnetic resonance imaging (MRI) or computed tomography (CT). The lesion was well defined in all patients. The diameter of the hematoma ranged from 7 to 25 cm, with a mean of 13.5 cm. In patients who underwent MRI, the lesion had a heterogeneous low to intermediate signal on T1-weighted imaging; the intermediate signal intensity was higher than that of skeletal muscle. T2-weighted imaging demonstrated low to high signal intensity. The MRI findings were consistent with those of typical hematomas. A pseudocapsule was characteristic of all chronic expanding hematomas in this series; the pseudocapsule was characterized by low signal intensity on T1- and T2-weighted images (Figures 1 and 2). In patients who underwent CT, the CT value of the lesion was lower than that seen in muscle, while the CT values for the pseudocapsule and any septum were the same as muscle (Figure 3). Cystic features were seen in 5 of the 7 patients.

All patients underwent lesion resection that included the pseudocapsule. The diagnosis of hematoma was pathologically confirmed in the resected material, and the pathologic findings were consistent in all patients. The resected specimens showed organizing hematomas with peripheral neovascularization and papillary endothelial hyperplasia. The cyst wall was thickened with fibrous connective tissue with inflamed granulation tissue. It also contained foreign body-type multinucleated giant cells and aggregates of histiocytes. In the 6 subcutaneous lesions, the adjacent fascia was adherent to the pseudocapsule, and it was necessary to resect this fascia. No patients experienced complications from this fascia resection. In 3 of these 6 patients, the overlying skin was also adherent and required partial resection in order to avoid skin necrosis. In the patient with the deep-seated lesion, the common peroneal nerve was completely adhered to the pseudocapsule. The nerve was not able to be identified and thus was inadvertently resected. This patient experienced the drop-foot phenomenon from absence of the common peroneal nerve after surgery. There was no improvement after 1 year of follow-up. The duration of follow-up ranged from 12 to 77 months, with a mean of 36.8 months. No patients experienced recurrence during the follow-up period.

## 3. Discussion

The current case series describing chronic expanding hematoma in the extremities is male-dominant, with a mean age at time of resection of 65.6 years. The duration of the hematoma's presence ranged from 3 to 30 years, with a median duration of 10 years. All chronic expanding hematomas occurred in the lower extremities, predominantly in the subcutaneous tissue. These findings all seem to be characteristic of chronic expanding hematoma.

Chronic expanding hematoma is a mixture of old and new blood with time-related changes present. The histological features are reportedly a mixture of blood breakdown products, granulation tissue with capillary ingrowth, and inflammatory tissue [3]. Corresponding to the various histological findings, MRI shows heterogeneous low to intermediate signal intensity. A pseudocapsule with low signal intensity on T1- and T2-weighted imaging is characteristic of chronic expanding hematomas. Histologically, the pseudocapsule is composed of fibrous tissue with hemosiderin deposits and iron-laden macrophages [1, 4]. In the current series, the preoperative imaging modality was CT in 2 patients; these patients were seen early in the study period, when the superiority of MRI was not recognized. Although a characteristic pseudocapsule is seen on CT, the use of MRI is preferred for an accurate imaging diagnosis of chronic expanding hematoma.

Although the MRI diagnosis of chronic expanding hematoma is relatively easy, the findings are similar to those of hemorrhagic soft-tissue sarcomas [2, 5]. Careful examination of every MRI section to detect a nonhemorrhagic portion of a possible neoplasm is necessary. Not only imaging results but also clinical information (e.g., speed of growth) is important in the diagnosis of chronic expanding hematoma. The continuous, long-term endothelial stimulation in a chronic hematoma could contribute to neoplastic transformation. A previous report noted that angiosarcoma can arise from chronic expanding hematomas at the periphery of the pseudocapsule. Therefore, a thick pseudocapsule wall could be a finding either of malignant transformation or of sudden or uncontrolled hematoma enlargement [6].

A possible association with trauma has been reported [2], but only 3 of 7 patients in our series had a history of trauma. Interestingly, anticoagulation medication was taken by only 1 patient, even though anticoagulation medication is a risk factor for bleeding. Formation of a fibrous pseudocapsule seems to be necessary for the creation of a chronic expanding hematoma; therefore, a normal coagulation status might be favorable to its formation.

The ideal treatment for chronic expanding hematoma is complete removal, including the pseudocapsule [7]. In a previous report, follow-up revealed that 2 of 9 chronic expanding hematomas recurred after marginal excision [8]; however, none of our 7 patients experienced recurrence. However, complete removal is reportedly difficult in patients with thoracic lesions because of abundant new vascularization beneath the pseudocapsule and the presence of fibrous adhesions to the chest wall [9]. In our patients with subcutaneous hematomas, the adjacent fascia was adherent in all, and skin resection was required in half. However, the removal of the fascia, with or without the skin, seemed not to affect subsequent functional ability. On the other hand, in the patient with a deep thigh lesion, the adjacent nerve was adherent to the pseudocapsule and dissection of the nerve was not possible. One report details resection of a chronic expanding hematoma, leaving the pseudocapsule in situ, as adequate treatment for a thoracic lesion [10]. In our patient with deep thigh involvement, leaving the pseudocapsule in situ could have avoided the subsequent nervous complications. However, because of the massive cystic features present in this patient's lesion, removal of the inside of the lesion, without removing the pseudocapsule, may not have been sufficient for treatment.

This study has a few limitations. First, the study was based on a small number of patients (*N* = 7), and only 1 case with a deep-seated hematoma was included. However, in all 7 cases, the adherent nature of the pseudocapsule was a consistent finding. Second, the follow-up periods were relatively short; the mean duration of follow-up was 36.8 months, and the longest follow-up period was 77 months. None of the patients recurred during the follow-up period. A longer follow-up period is needed for determining the recurrence rate.

In conclusion, chronic expanding hematoma of the extremities predominantly occurs in the subcutaneous tissues of the lower extremities. The clinical problem of adhesions to the surrounding tissue should be recognized during resection, especially in patients with deep-seated lesions.

## Figures and Tables

**Table 1:** Summary of chronic expanding hematomas in the extremities.

Case	Age (yr)/gender	Location	Shallow/deep	Size	Duration	Trauma	Anticoagulation	Cystic findings	Adherent tissue
#1	67/M	Knee	Shallow	7 cm	10 years	−	+	−	Fascia
#2	69/F	Thigh	Deep	25 cm	10 years	−	−	+	Nerve, fascia
#3	61/M	Thigh	Shallow	9 cm	3 years	+	−	+	Skin, fascia
#4	68/M	Thigh	Shallow	17 cm	23 years	+	−	−	Skin, fascia
#5	54/M	Thigh	Shallow	20 cm	15 years	+	−	+	Fascia
#6	61/M	Knee	Shallow	10 cm	30 years	−	−	+	Skin, fascia
#7	79/M	Knee	Shallow	7 cm	9 years	−	−	+	Fascia

M: male; F: female.
